# Cottage Hospitals as Sanatoria

**Published:** 1912-08-31

**Authors:** 


### COTTAGE HOSPITALS AS SANATORIA.

Over the signature "Governor" there appears a letter in the *Darlington Northern Echo* advocating that some of the local cottage hospitals should be used as sanatoria, since much of the present work of these institutions is overlapping the greater and more skilled work of the large city hospitals, thereby causing excessive waste. The example given is that of the Alnwick Infirmary (12 beds), which is stated to have recently been empty for four weeks, while at the same time there were five patients from its district in the Newcastle-upon-Tyne Infirmary. The writer urges that these cottage hospitals, many of which are ideally situated, could be partly used for consumptive patients, with two small wards reserved for urgent local surgical or accident work, the arrangement to be subsidised out of the new county funds to be provided by the Government; and the objections of keen operating general practitioners to be met by "giving them greater facilities to attend operations on their patients at the city hospitals." There is an apparent plausibility in the suggestion which may very likely make it a popular cry now that the public is getting somewhat alarmed at the present lack of means to supply the much-advertised sanatoria benefit.

But the weak point of this scheme is in the last clause. It is hardly fair that the difficulties of operating in rural practice (and the necessity for operating is explicitly recognised in the letter) should be increased. It is no answer to say that emergencies are provided for. If you throw upon a man the onus of undertaking emergency surgery, it is only equitable and only wise to let him keep his hand in to some extent. From a practical point of view the whole matter turns on the number of rural cottage hospitals which have been empty for any time recently. For it is a fact that in the picturesque little town under the shadow of Hotspur's Castle the infirmary was empty for a while. If this state of affairs is at all general, why then the idle beds should certainly be used for some good purpose. But as regards provision of sanatorium benefit, they can never be anything but what Mr. Hurry B. Jabberjee might have called a "stop-gap drop in the bucket." And then the difficulty might arise again of patients preferring central institutions. There ventilation would be better, accommodation better, there would be more company—let alone the advantages of care by a specially trained medical man who could use tuberculin and undertake laryngology. But we doubt whether as a fact it will come to this. Our cottage hospitals as a class are kept pretty busy, and with the growth of new centres of population there are not many districts of twenty miles radius over-supplied with hospital beds or destitute of competent operators. From Alnwick to Newcastle is a straight run on that best of highways, the Great North Road, it is true, and nowadays almost anyone can get the loan of a car to take a sick relative to hospital; nevertheless, there are enough inevitable discomforts in invalid transport, one would have thought, to have caused full utilisation of a dozen beds. There is also perhaps a fatal fascination in the title. "Cottage hospital" suggests the sylvan surroundings that sanatoria require, at any rate to the lay mind. This, coupled with the temporary emptiness of the neighbouring small hospital, has produced the present proposal, the author of which, however, has most incautiously generalised from local conditions, and assumed much too hastily that cottage hospitals in general have beds to spare for this purpose.

Another objection, though perhaps a less serious one to "Governor's" suggestion, is that of expense. It is much more costly *pro rata* to feed and nurse patients in small institutions than in large ones; administration charges of all kinds are heavier, and efficiency is only too likely to be sacrificed. In a large sanatorium a certain percentage (which varies very slightly indeed) of the patients can be relied on to be in bed, a certain percentage to be up but resting, a certain percentage to be ambulant. Thus many administrative details can be foreseen pretty accurately and provided. But in a small institution such as a cottage hospital the law of averages has no scope, and wide fluctuations in requirements are probable.

